# Contrasting effects of nitrogen and phosphorus addition on soil respiration in an alpine grassland on the Qinghai-Tibetan Plateau

**DOI:** 10.1038/srep34786

**Published:** 2016-10-10

**Authors:** Fei Ren, Xiaoxia Yang, Huakun Zhou, Wenyan Zhu, Zhenhua Zhang, Litong Chen, Guangmin Cao, Jin-Sheng He

**Affiliations:** 1 Key Laboratory of Adaptation and Evolution of Plateau Biota, Northwest Institute of Plateau Biology, Chinese Academy of Sciences, 23 Xinning Rd., Xining, 810008, China; 2 University of Chinese Academy of Sciences, 19A Yuquan Rd., Beijing, 100049, China; 3 State Key Laboratory of Plateau Ecology and Agriculture, Qinghai University, 251 Ningda Rd., Xining, 810016, China; 4 Qinghai Academy of Animal and Veterinary Sciences, 1 Weier Rd., Xining 810016, China; 5 Department of Ecology, College of Urban and Environmental Sciences, and Key Laboratory for Earth Surface Processes of the Ministry of Education, Peking University, 5 Yiheyuan Rd., Beijing 100871, China

## Abstract

High soil organic carbon content, extensive root biomass, and low nutrient availability make alpine grasslands an important ecosystem for assessing the influence of nutrient enrichment on soil respiration (SR). We conducted a four-year (2009–2012) field experiment in an alpine grassland on the Qinghai-Tibetan Plateau to examine the individual and combined effects of nitrogen (N, 100 kg ha^−1^year^−1^) and phosphorus (P, 50 kg ha^−1^year^−1^) addition on SR. We found that both N and P addition did not affect the overall growing-season SR but effects varied by year: with N addition SR increased in the first year but decreased during the last two years. However, while P addition did not affect SR during the first two years, SR increased during the last two years. No interactive effects of N and P addition were observed, and both N addition and P addition reduced heterotrophic respiration during the last year of the experiment. N and P addition affected SR via different processes: N mainly affected heterotrophic respiration, whereas P largely influenced autotrophic respiration. Our results highlight the divergent effects of N and P addition on SR and address the important potential of P enrichment for regulating SR and the carbon balance in alpine grasslands.

Soil respiration (SR) represents the second-largest flux in the terrestrial carbon cycle between soil and the atmosphere. SR dynamics and its response to increasing global change factors are of primary relevance to regional and global carbon storage, and the feedbacks between changes in climate and terrestrial ecosystems^1^,^2^,^3^,^4^. SR consists of both autotrophic respiration (SR_A_) and heterotrophic respiration (SR_H_). SR_A_ results from roots, mycorrhizae and other rhizospheric microorganisms, and SR_H_ results from free-living soil and non-rhizospheric microorganisms^2^,^5^,^6^,^7^. The response of SR to biotic and abiotic factors resulting from anthropogenic global change is mainly mediated by SR_A_ and SR_H_ individually or together and, both directly and indirectly^8^,^9^. The responses of SR_A_ and SR_H_ to global change drivers may differ^10^, and the difference between net primary production and SR_H_ determines the net ecosystem carbon balance^4^. Therefore, partitioning the autotrophic and heterotrophic components of SR is essential for assessing underground carbon fluxes and the carbon balance of terrestrial ecosystems, and for providing mechanistic insights into the response patterns of SR to global change.

Because of the strong coupling among the carbon, nitrogen (N) and phosphorus (P) cycles^11^, detailed information about the influence of N and P on SR is essential for improving our knowledge of carbon dynamics. Understanding how SR responds to increased N and P availability is particularly important because terrestrial primary productivity is generally co-limited by N and P^12^, while human activity has increased N and P inputs to ecosystems. Global warming and changes in precipitation patterns enhance atmospheric N and P deposition alongside more fertilizer use^13^,^14^,^15^. The effects of N enrichment on SR have been well documented in forest ecosystems^2^,^15^,^16^,^17^,^18^,^19^, temperate grasslands^10^,^20^,^21^ and other ecosystems and regions. However, the results are highly controversial and may be positive^22^,^23^,^24^,^25^, negative^16^ or neutral^15^,^26^,^27^,^28^,^29^. This is potentially due to differences in ecosystem types, N addition rates, and duration of fertilization periods. For example, N addition has been observed to influence SR differently during different time periods, even within the same ecosystem^21^,^25^,^30^.

However, compared to the large numbers of studies exploring the influence of N on SR, the understanding of how increasing P inputs affect SR and thus carbon storage is limited^31^,^32^. Although some studies have compared the combined effects of N and P additions on SR, data regarding the responses of SR to P addition, alone or in combination with N, over a relatively long period of time in nutrient-limited regions, such as the alpine grasslands on the Qinghai-Tibetan Plateau, are almost entirely lacking.

Alpine grassland is the typical vegetation type and the main pasture on the Qinghai-Tibetan Plateau. The Qinghai-Tibetan Plateau is one of the most understudied regions with regard to SR^33^, and is ideal for comparing the responses of SR to N and P enrichment because the soils of alpine grassland ecosystems contain large amounts of organic C (7.4 Pg within the top 100 cm of soil^34^). Since this region experiences low temperatures that suppress the activity of soil biota and consequently litter decomposition and soil organic matter mineralization, plant and microbial growth in this region are strongly limited by available soil nutrients^35^ (especially N and P). Thus, plants that grow on the Qinghai-Tibetan Plateau have large root biomass with high partitioning of photosynthates to this underground biomass. Therefore, as the main source of belowground-related carbon flux, SR may be sensitive to the amelioration of nutrient availability. Small changes in SR in response to anthropogenic nutrient enrichment can potentially influence carbon stocks and subsequent global change.

The Qinghai-Tibetan plateau is experiencing “much greater than average” global changes, such as increased atmospheric N deposition^36^,^37^ and local climate warming^14^. N deposition in the Qinghai-Tibetan Plateau is clearly increasing, although the amount of N deposition remains relatively low compared to central China^36^,^37^. For instance, Jia *et al*.^38^ reported that wet N deposition in the Qinghai-Tibetan Plateau increased by 27.6–41.5%, whereas the mean wet N deposition across China from the 1990s to the 2000s increased by 25%. Moreover, the rapid increase in the surface temperature on the Qinghai-Tibetan plateau could stimulate the soil organic matter and plant litter decomposition rates, which may induce higher N and P availability in the grassland ecosystems in this region^14^. Therefore, it is important to quantify SR in alpine ecosystems and understand the drivers and potential impacts of future global change.

However, to our knowledge, only a few studies have focused on the influence of N on SR in alpine grasslands, and the results were varied, giving both positive and neutral responses. For example, in a two-year field experiment, Fang *et al*.^39^ reported that N addition had a positive effect on SR. However, in an incubation experiment using samples obtained from the same region, Song *et al*.^35^ found that N addition had no effect on basal SR. The influence of P on SR and the partitioning of the autotrophic and heterotrophic components of SR in this region have not been studied. Therefore, it remains unclear how N and P enrichment due to global changes affect SR and its components in alpine grasslands, and whether the response of SR to N is distinct from the response of SR to P addition.

To evaluate the effects of N and P addition on SR, we conducted a four-year (2009–2012) experiment of N and P addition in an alpine grassland in the northeast region of the Qinghai-Tibetan Plateau. Our research specifically addressed the following aims: 1) to evaluate the effects of N and P addition on SR and its components, 2) to assess the temporal variation in the response of SR to N and P addition.

## Results

### Microclimate and seasonal variations in soil respiration

**Figure 1:**
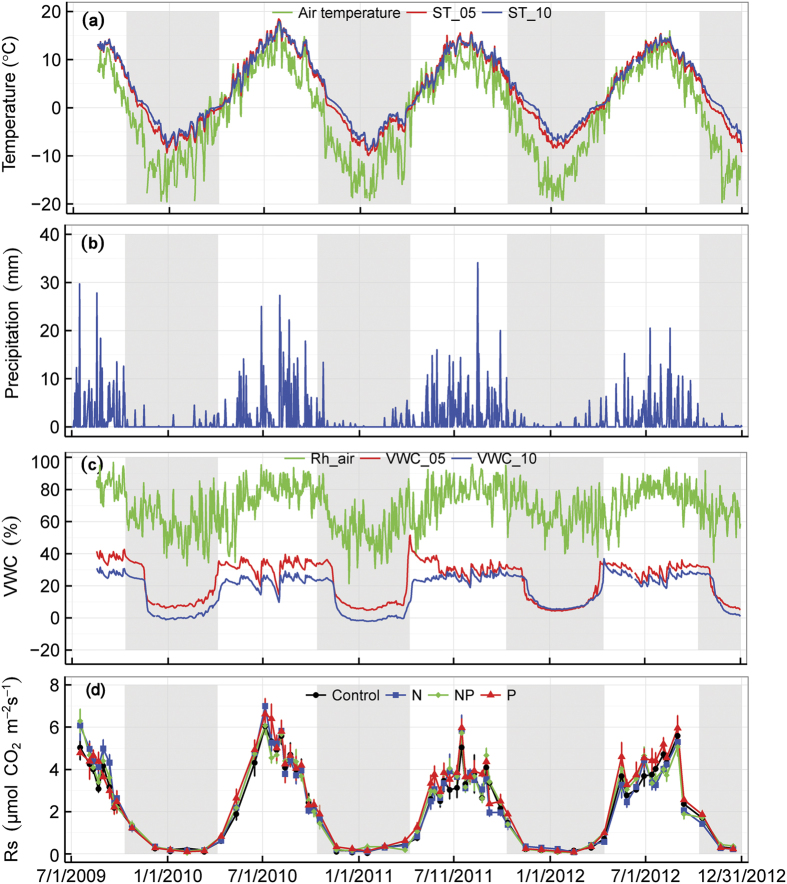
Seasonal and annual variation in (**a**) air temperature and soil temperature at a depth of 5 cm (ST_5) and soil temperature at a depth of 10 cm (ST_10); (**b**) precipitation; (**c**) relative humidity (Rh_air), volumetric soil moisture at 5 cm depth (VWC_5) and volumetric soil moisture at 10 cm depth (VWC_10); and (**d**) soil respiration rates under different treatments from July 1^st^ in 2009 to September 30^th^ in 2012. Shaded areas represent the non-growing season.

The seasonal patterns of temperature, precipitation, soil water content and SR fluxes are shown in Fig. 1. From 2009 to 2012, the mean annual air temperature ranged from −0.81 to −1.82 °C, and annual precipitation from 350.6 to 501.3 mm. Soil temperature and moisture were neither extremely high nor low during the growing season; mean soil temperatures at a depth of 5 cm were 12.01, 11.48, 10.20 and 9.88 °C (Fig. 1a), and mean soil moisture at the same depth were 37.0, 31.5, 30.5, 30.5 V/V% (Fig. 1b) in 2009 (August 17^th^-September 30^th^), 2010, 2011 and 2012, respectively. The total amount of precipitation during the growing season (May-September) was 444.6 mm in 2009, 403.8 mm in 2010, 447.5 mm in 2011 and 319.2 mm in 2012 (Fig. 1c).

The seasonal pattern of SR generally followed the variations in soil temperature during the four years of the study, with higher values during the middle of the growing seasons and lower values during the earlier and later periods (Fig.1d). Inter-annual variability in SR was observed (Table 1), with 2010 having the highest seasonal mean SR (4.33 μ mol m^−2^ s^−1^), the highest mean soil temperature (11.7 ^o^C), and the highest mean soil moisture content (31.5%, VWC), occurring from May to September.

### Soil respiration during the growing season and its component

The nutrient addition treatments did not affect SR outside the growing season (data not shown). Therefore, in this study we only present the effects of N and P on the SR rates during the growing season.

**Figure 2:**
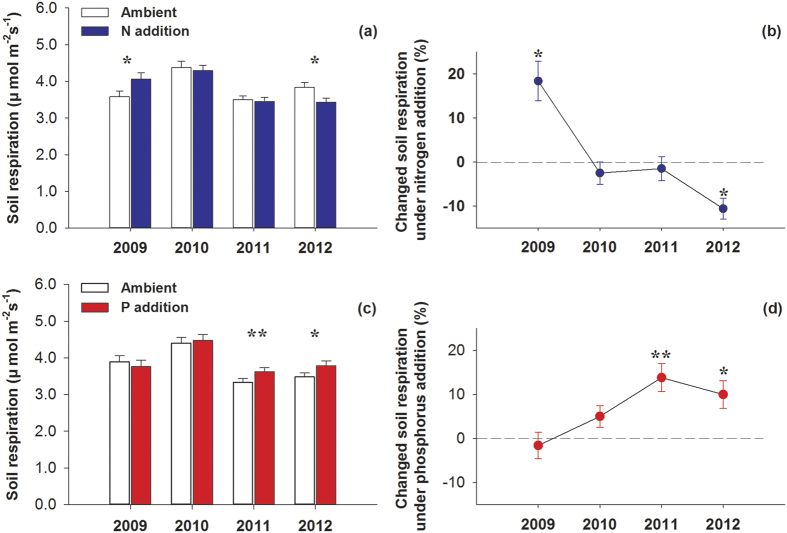
Effects of nitrogen and phosphorus addition on soil respiration (**a,c**) and changes (%) in soil respiration (**b,d**) during the growing seasons from 2009 to 2012. ^*^P < 0.05, ^**^P < 0.01. Error bars indicate the standard errors.

Overall, nutrient addition had no significant effect on SR during the growing season and no interactive effects of N and P addition were observed. However, the response of SR to N addition varied during the measurement periods, as indicated by a significant interaction effect between N addition and measurement date on SR (Table 1). When analyzing fertilization effects each year, N addition significantly enhanced SR by 18.4% (*P* = 0.028) in 2009, had no significant effect in 2010 and 2011, and reduced SR by 10.2% (*P* = 0.005) in 2012 (Table 2; Fig. 2a,b). Unlike the effects of N addition, P addition hardly affected SR during the first two years but significantly stimulated SR by 13.8% (*P* = 0.022) and 9.99% (*P* = 0.015) in 2011 and 2012, respectively (Table 2; Fig. 2c,d).

**Figure 3:**
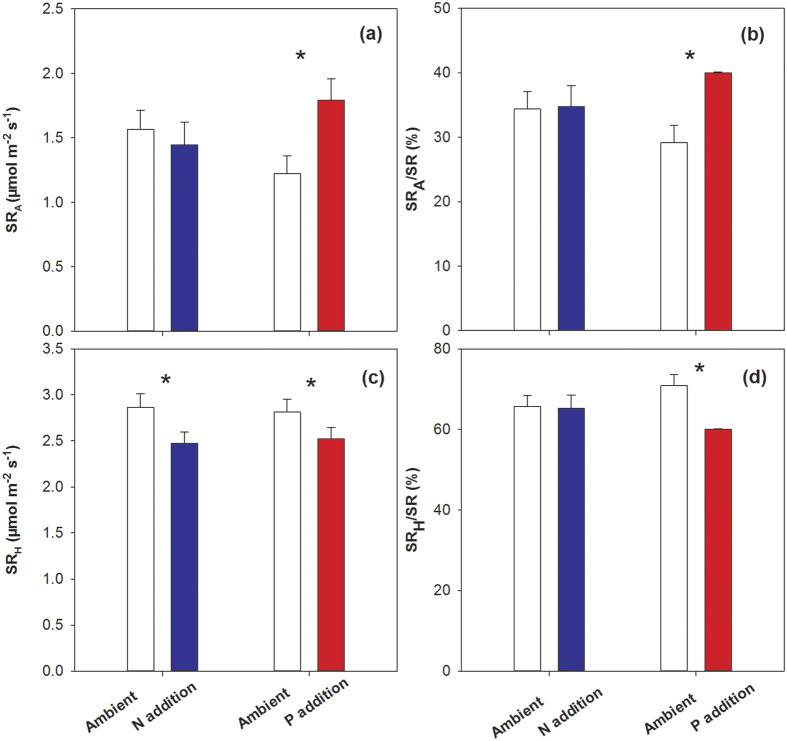
Effects of nitrogen and phosphorous addition on (**a**) autotrophic respiration (SR_A_), (**b**) the ratio of SR_A_ to soil respiration (SR_A_/SR), (**c**) heterotrophic respiration (SR_H_) and (**d**) the ratio of SR_H_ to soil respiration (SR_H_/SR) in 2012. ^*^P < 0.05. Error bars indicate the standard errors.

For SR_A_, P addition significantly increased the seasonal mean SR_A_ (by 46.56%, *P* = 0.003, Table 3; Fig. 3a) and its contribution to total SR (SR_A_/SR, by 27.09%, *P* = 0.002, Table 3; Fig. 3b) in 2012. However, only a nonsignificant trend toward lower SR_A_ was observed in response to N addition, and no interactions between N and P effects on SR_A_ and SR_A_/SR were observed (Table 3; Fig. 3a,b).

For SR_H_, both N addition and P addition significantly reduced the seasonal mean SR_H_ (by 13.77% (*P *< 0.001) and by10.30% (*P* < 0.001) in 2012, respectively (Table 3; Fig. 3c), while no interactions between N and P effects on SR_H_ were observed. In addition, only P addition significantly decreased the contribution of SR_H_ to the total SR (SR_H_/SR, by 15.28%, Table 3; Fig. 3d).

### Biotic and abiotic factors

No effects of nutrient addition on soil temperature and soil moisture were found across the years of this study (Supplementary Table 1). Overall, the rate of SR was exponentially related to soil temperature (*P* < 0.01, *R*^*2*^ = 0.488, Supplementary Fig. 1b). The range in soil moisture was large; however, the overall relationship between SR and soil moisture was negative (*P* < 0.01, *R*^*2*^ = 0.083, Supplementary Fig. 1b).

**Figure 4:**
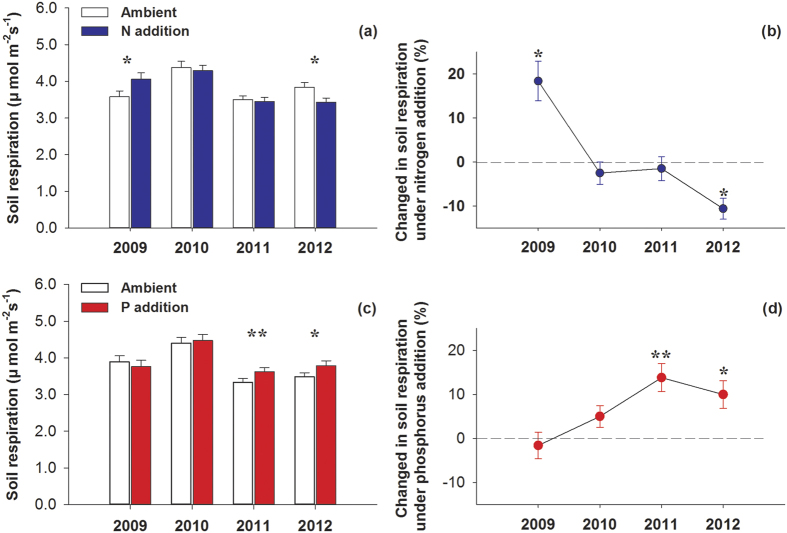
Effects of nitrogen and phosphorus addition on (**a**) aboveground biomass (AGB), (**b**) belowground biomass (BGB), (**c**) root nitrogen content (Root [N]) and (**d**) root phosphorus content (Root [P]) in 2012. **′***P* < 0.1, ^*^P < 0.05, ^**^P < 0.01, ^***^ *P *< 0.001.

Both N addition and P addition significantly increased aboveground biomass (Fig. 4a), while the addition of only P marginally increased belowground biomass in 2012 (Fig. 4b). N addition slightly increased root N content compared with the control plots (Fig. 4c), and P addition significantly increased root P content by a factor of almost 2.5 compared with the control plots in 2012 (Fig. 4d).

## Discussion

According to a study using automatic continuous measurement of SR in this region^40^ and our results of the inter-annual dynamics of SR (Fig. 1d), the non-growing season SR rates were low and accounted for only 11.8–13.2% of the total annual SR. In this study we only discuss the effects of N and P on the SR rates during the growing season. After adding N and P to an alpine grassland on the Qinghai-Tibetan Plateau for four years, we found that both N and P addition changed the SR rates during the growing seasons. However, the effects of adding N and P were contrasting and varied dramatically over the four years.

### Effects of N addition

Our study shows that the response of SR to N addition varies over time: N addition stimulated SR rates during the growing season in the first year of the experiment, had no effect in the following two years, and decreased the SR rates in the fourth year. The divergent responses of SR to N addition over time in our study illustrate the importance of conducting longer-term field studies to assess the impacts of N addition, such as those resulting from high rates of atmospheric deposition^41^. The timing of the alpine grassland ecosystem responses to N addition agreed with the results obtained in semiarid temperate steppes. That is, different N addition rates increase SR during the first year, and have no effect or even decrease SR during subsequent years^10^,^42^,^43^. Similar temporal responses to long-term N addition have been observed in forest ecosystems, in which SR increased during the first year of N addition, and decreased after many years of treatment, in Harvard Forest^26^,^44^.

The effects of N addition on SR depend on the responses of SR_A_ and SR_H_ to N^2^,^10^,^23^,^29^,^45^. In an alpine grassland, Jiang *et al*.^46^ found that both plant growth and microbial activity were generally N-limited, but the ability of plants to capture soil inorganic N was much stronger than that of soil microorganisms^47^. When N was initially added, increased N availability resulted in increased plant growth, microbial activity and plant biomass^26^,^44^.Therefore, the decomposition of litter and SOM is enhanced by increasing the quantity of litter input or by elevating microbial activity. Thus, the combined stimulation of both SR_A_ and SR_H_ become more likely, increasing overall SR during the early stages of the experiment. This speculation is consistent with our results that indicate that N addition stimulated SR rates during the 2009 growing season (Figs 1d and 2a,b).

However, it is suggested that chronic N addition to N-limited forest soils should initially stimulate soil microbial activity and then result in a carbon-limited state after the microbial demand for N is satisfied^48^. This amendment-induced C limitation to microorganisms would result in the suppression of microbial activity and corresponding decreases in SR_H_ and SR when N is added over a long period^29^,^41^,^45^. Moreover, the bacteria responsible for decomposing cellulose are N-limited and generally stimulated by N input^15^(which is helpful for interpreting the initial stimulation of SR during the early stages of N addition when labile carbon is not depleted).Whereas, the oxidative activities associated with recalcitrant litter or SOM are usually depressed by N because the fungi responsible for decomposing recalcitrant materials are generally adapted to low N conditions^4^. Additionally, an investigation near our experiment site indicated that soil particulate organic carbon (labile carbon) decreased after three years of N addition^49^, proving that microbial activity is enhanced at the beginning of N treatment and that more labile carbon is depleted. Thereafter, the ratio of labile to recalcitrant carbon decreased, with the N input probably stimulating microbial activity before decreasing it 26,48. Our study suggests that N addition decreased the SR_H_ after four years of N addition (Fig. 3); which agreed with the results of other studies^24^,^16^,^44^,^45^. In conclusion, we suggest that N addition stimulates SR_H_ before decreasing it in alpine grasslands.

**Figure 5:**
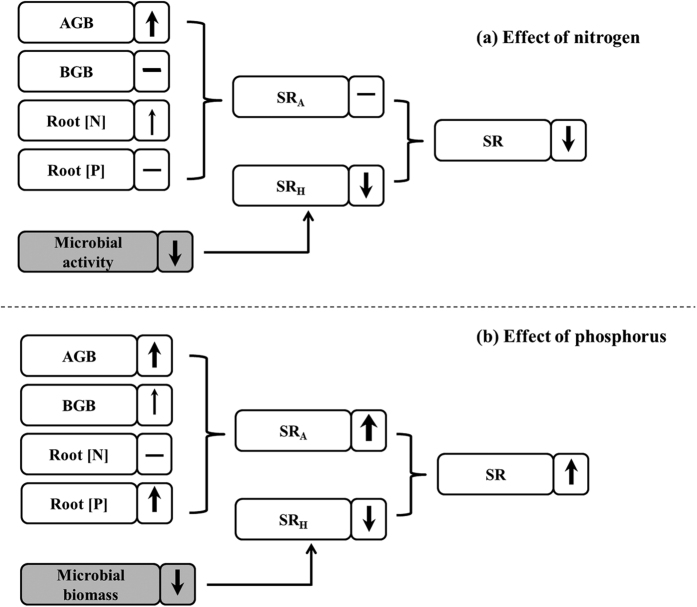
Model of the effects of nitrogen (**a**) and phosphorus (**b**) addition (over four years) on soil respiration. The thickness of the arrows represents the strengths of the treatments; the solid arrows indicate where data were measured, and dashed arrows indicate where data were not measured in this study. Up arrows represent positive effects, and down arrows represent negative effects, while dashes represent neutral effects. The white boxes are the results from the present study, and the grey boxes are the results from previous studies.

Besides, N addition also influences SR_A_ by affecting belowground biomass and root N content^4^. Generally, the down-regulation of SR_A_ rates occurred in response to increased N availability^23^. Although this reduction is seldom relative to the nutrient-induced suppression of SR_H_, the effect when combined with that of SR_H_ may significantly contribute to the overall decline in SR under N addition (Fig. 3). Despite N limitation for plant growth in alpine ecosystems, no significant effects of N addition on belowground biomass were detected in 2012 in this study (Fig. 4b). We did not find any difference in root N content (Fig. 4c) between the N addition and control plots, either. These results indicate that SR_A_ is probably not affected by N addition in alpine grasslands. Overall, the temporal variation of the response of SR to N addition in alpine grasslands on the Qinghai-Tibetan Plateau may largely result from the temporal variations in the responses of microbial activity to N addition (Fig. 5a).

### Effects of P addition

In contrast to the effects of N addition, this study shows that P addition has a positive effect on SR in alpine grassland ecosystems on the Qinghai-Tibetan Plateau. Although fewer studies have examined the response of SR to P than to N addition, several studies have reported positive effects of P addition on SR in different ecosystems^32^,^50^. However, inconsistent responses of microbial activity to P enrichment have been reported^51^,^52^,^53^. Our study shows that P addition has a negative effect on SR_H_, which is consistent with a study conducted in the same region in which P addition reduced the abundance of arbuscular mycorrhizal (AM) fungi^50^.

Although microbial biomass-associated SR_H_ was significantly reduced by P addition, belowground biomass tended to increase, SR_A_ and total SR was stimulated by P addition in 2012 (Figs 2 and 3). It is obvious that the increase of SR in response to P addition has resulted from the positive responses of SR_A_ to P enrichment, which indicated that the responses of SR_H_ and SR_A_ were unsynchronized. Higher SR_A_ and lower SR_H_ following P addition resulted in significant decreases in the contribution of SR_H_ to SR. P is an important limiting factor to plant growth in this region. Both aboveground and belowground biomass were stimulated by P addition in the present study in 2012 (Fig. 4a,b), which is consistent with investigations in this region^54^ and with studies in other regions^55^. Consequently, the SR_A_ rate was stimulated by P addition based on the increased root biomass. Moreover, the root P content was enhanced by a factor of nearly 2.5 by P addition in our study. Given the important role of P in all physical processes, especially nucleic acid synthesis, membrane synthesis, energy generation and enzyme activation/inactivation^56^, we speculate that the high P content of the roots is probably related to high root maintenance respiration (*i.e*., specific root respiration). In conclusion, the stimulation SR following P addition in the investigated alpine grassland ecosystem results from the enhancement of SR_A_ due to the positive effects of P addition on belowground biomass and root P content. In addition, because of the delayed response of plants to P addition, the tendency of the stimulation of SR by P addition was not significant until the third year (Fig. 5b). Nevertheless, more thorough research that focuses on the effects of P addition and on microbial activity and decomposition rates of litter and SOM should be carried out.

### Conclusions and implications

Collectively, this study shows the contrasting responses of SR to N and P addition in an alpine grassland on the Qinghai-Tibetan Plateau. The influence of N addition varied with time after treatments: SR was stimulated, remained stable, and then decreased. In contrast to N addition, P addition stimulated SR but this was not significant until the third year. These contrasting responses may be explained by the different responses of plant-associated SR_A_ and microbial-associated SR_H_ to N and P enrichment. The year-to-year variations in the SR responses to N addition may largely stem from the temporal variations of responses of microbial activities to N addition (Fig. 5a), whereas the positive response of SR to P addition may result from the stimulation of P to plant productivity (Fig. 5b). Moreover, both N and P addition reduced the SR_H_ in 2012. Given that only SR_H_ results from SOM decomposition, our results suggest that both N and P addition are likely to enhance soil carbon sequestration in the alpine grasslands of the Qinghai-Tibetan Plateau. That is to say, alpine grassland ecosystems may act as carbon sinks with further increases in nutrient availability.

In order to predict and quantify the response of SR and subsequent soil carbon sequestration to nutrient addition, and to better understand the mechanisms of these changes, we need long-term research on SR, SR_A_, SR_H_ and ancillary information, especially regarding fine root biomass, soil pH changes, soil organic carbon composition and plant and microbial community composition.

## Materials and Methods

### Study site

The study site is located approximately 2 km northeast of the Haibei Alpine Meadow Ecosystem Research Station^57^ of the Northwest Institute of Plateau Biology, Chinese Academy of Sciences. The site is on alpine grassland in the northeastern region of the Qinghai-Tibetan Plateau, located at 37°37′ N, 101°12′ E, in Qinghai Province, China. The terrain of the study site is flat and open, an elevation of 3,250 m and with uniform vegetation that has never been fertilized. The experimental site has been overgrazed in the past, but was fenced and has only been grazed in winter and early spring (October to April in the following year) since 2001. The climate is dominated by the southeast monsoon and the Siberian high-pressure system and classified as continental monsoon. This is characterized by strong solar radiation with long, cold non-growing seasons (from January to April and from October to December) and short, cool growing seasons (from May to September). Detailed climate information for the study site is available elsewhere^40^,^57^.The soils have a clay loam texture with a mean thickness of 0.65 m, classified as Mat-Cryic Cambisols (alpine meadow soil) according to the Chinese National Soil Survey and Classification System^58^, or as Gelic Cambisols according to the FAO classification system. The main soil characteristics of the study site are shown in Supplementary Table 2.

The plant community at the experimental site is dominated by *Kobresia humilis*, *Stipa aliena*, *Elymus nutans and Festuca ovina*. Abundant species include *F. rubra*, *Deyeuxia flavens*, *Koeleria litvinowii* var. litvinowii, *Saussurea pulchra*, *Gentiana straminea*, *Gentiana lawrencei* var. farreri, *Oxytropis ochrocephala*, *Medicago archiducis-nicolai* and *Tibetia himalaica*.

### Experimental design

The experimental design followed the standard protocols of Nutrient Network (NutNet; http://nutnet.umn.edu). In mid-May 2009, we fenced an experimental area of 1 ha to prevent grazing disturbance. We randomly assigned 24 plots of 6 × 6 m to 4 treatments with 6 replicates (blocks) in a complete randomized block design. The blocks were separated by a 2-m-wider buffer zone, and the plots within each block were separated by a 1-m-wider buffer zone to minimize disturbance from neighboring treatments. Each plot was divided into three subplots with observations of phenology in subplot 1 (3 m × 3 m), and measurements of carbon fluxes in subplot 2 (3 m × 3 m), and measurements of plant biomass and soil characteristics in subplot 3 (6 m × 3 m). The four treatments consisted of the following: (1) Control, no fertilizer was added; (2) N addition (in the form of urea, 100 kg N ha^−1 ^year^−1^); (3) P addition (in the form of triple superphosphate, 50 kg ha^−1^ year^−1^); and (4) NP addition (combined addition of N and P in the same amounts as the solo treatments). In the fertilized treatments, pelletized fertilizer was evenly distributed by hand onto the plots after sunset (for higher moisture) on July 15^th^ in 2009, July 5^th^ in 2010, July 27^th^ in 2011 and June 22^nd^ in 2012.

### Measurement protocols

#### Soil respiration and heterotrophic component

SR measurements were conducted using a polyvinyl chloride (PVC) collar in each plot during 2009 and 2012. We used a portable soil CO_2_ flux system (Li-8100, Li-COR, Inc., Lincoln, NE, USA) attached to a soil CO_2_ flux chamber (Li-8100-02, Li-COR Inc.). In each plot, one PVC collar (20 cm inside diameter and 7.5 cm height, short collar) was placed at least 30 cm away from the edge of subplot 2 and inserted into the soil to a depth of 5 cm in July 2009. We measured the SR in the short collar three to four times per month during the growing seasons (May-September) and once per month during non-growing seasons. Living plants inside the collars were clipped and removed at least one day before the measurements to eliminate aboveground plant respiration. The collars were moved to adjacent areas (still in subplot 2) in mid-May of each year to avoid SR underestimation from the suppression of root growth by collar insertion and periodic clipping.

Another PVC collar (20 cm inside diameter and 50 cm height, deep collar) was inserted into the soil to a depth of 47.5 cm in each plot to measure SR_H_ at the end of the growing season in 2010. The insertion of deep collars can cut off old plant roots and prevent new roots from growing inside the collars. We continued to clip plants inside the collar during the growing seasons of 2010 and 2011 to suppress living root growth inside the collar until the SR measured in these collars mainly resulted from heterotrophic respiration. The collars remained in place throughout the experiment. SR_H_ was measured once a month in the deep collar during the growing season (from May to August) of 2012.

The SR rates were calculated as the means of four plots for each measurement. SR_A_ was calculated as the difference between SR and SR_H_.

### Soil temperature and soil moisture

When SR was measured, the soil temperature (^o^C) at a depth of 5 cm was measured adjacent to the collar by using a thermocouple probe (Li-8100-201) connected to the Li-8100. Simultaneously, soil moisture (V/V %) was also recorded at a depth of 5 cm with a TDR moisture meter (IMKO, Ettlingen, Germany).

### Aboveground biomass and belowground biomass

The peak aboveground biomass was estimated annually by clipping living plants at the time of peak biomass. All living plants were harvested from a 0.25m × 0.25m quadrat in each subplot 3 in 2012. Harvested plants were oven-dried at 65 °C for at least 48 hours and weighed as aboveground biomass (AGB).

Roots were extracted using a soil corer (5 cm in diameter) at 10 cm intervals to a depth of 40 cm using an auger within the same quadrat after harvesting the aboveground biomass. In each plot, 3 cores were collected and mixed to form one sample and then immersed in water to remove the soil. After washing on a 0.5 mm mesh sieve, the roots were placed in paper bags, taken to the laboratory, oven-dried at 65 °C until a constant mass was achieved and then weighed to estimate belowground biomass (BGB).

### Elemental contents of the roots

After weighing the BGB, the root samples were grounded, passed through a 0.1 mm sieve and stored in paper bags. The root N content was analyzed using a 2400 II CHNS/O Element Analyzer (Perkin-Elmer, Boston, MA, USA), and the root P content was analyzed by using the method of colorimetry after acidifying the roots by digesting them with ammonium persulfate.

The measurements were performed in four blocks during the experiment period.

### Statistical analysis

Before statistical analysis, the SR rates from the four experimental blocks in each plot were averaged for each measurement date. Four-way ANOVA was used to analyze the main and interactive effects of year, N, P and the blocks on total SR and SR_H_. In all of the above analyses, the effects of the blocks were examined together with the treatments but were not presented or discussed because their effects were not significant. When the treatment by year interaction was significant, data were analyzed by year. Repeated measures analysis of variance (ANOVA) was used to analyze the effects of N, P and sampling date on SR for the four years, and on autotrophic respiration (SR_A_), heterotrophic respiration (SR_H_) and their contribution to total SR (SR_A_/SR, SR_H_/SR, respectively) in 2012. All statistical analyses were carried out using R version 3.1.1 (R development Core Team, 2013) or SPSS 20.0 (SPSS Inc., Chicago, IL, USA) and graphs were prepared using SigmaPlot 12.5 (Systat Software, Inc., Point Richmond, CA). Statistically significant differences were tested at *P* < 0.05 unless otherwise stated.

## Additional Information

**How to cite this article**: Ren, F. *et al*. Contrasting effects of nitrogen and phosphorus addition on soil respiration in an alpine grassland on the Qinghai-Tibetan Plateau. *Sci. Rep*. **6**, 34786; doi: 10.1038/srep34786 (2016).

## Supplementary Material

Supplementary Information

## Figures and Tables

**Table 1:** Summary of three-way analysis of variance (ANOVA) of nitrogen (N), phosphorus (P) and year (Y) on soil respiration from 2009 to 2012.

	d.f.	Mean square	F-values	*P*-values
Year (Y)	3	3.048	**20.81**	**< 0.001**
Nitrogen (N)	1	0.133	0.91	0.345
Phosphorus (P)	1	0.126	0.86	0.358
N × Y	3	0.917	**6.26**	**0.001**
P × Y	3	0.145	0.99	0.406
N × P	1	0.263	1.79	0.187

**Table 2:** F-ratios of the effects of nitrogen (N), phosphorous (P), measuring date (D) and their interactions on soil respiration (SR).

	Date (D)	Nitrogen (N)	Phosphorus (P)	N × P	N × D	P × D
2009	181.55^***^	6.84^*^	0.11^ns^	0.28^ns^	5.98^*^	0.10^ns^
2010	158.45^***^	0.25^ns^	0.08^ns^	2.04^ns^	1.00^ns^	1.62^ns^
2011	17.92^***^	0.39^ns^	7.71^**^	0.47^ns^	0.85^ns^	0.38^ns^
2012	113.10^***^	14.54^**^	9.43^*^	3.99^ns^	4.11^*^	3.5^*^

F-values and significance codes are shown. ***P < 0.001, **P < 0.01, *P < 0.05; ns indicates no significance.

**Table 3:** F-ratios of the effects of nitrogen (N), phosphorous (P), measuring date (D) and their interactions on autotrophic respiration (SR_A_), the ratio of SR_A_ to soil respiration (SR_A_/SR), heterotrophic respiration (SR_H_) and the ratio of SR_H_ to soil respiration (SR_H_/SR) in 2012.

	Date (D)	Nitrogen (N)	Phosphorus (P)	N × P	N × D	P × D
SR_A_	5.57^***^	0.33^ns^	7.74^**^	<0.001^ns^	0.45^ns^	0.38^ns^
SR_A_/SR	8.10^***^	0.01^ns^	9.14**	0.13^ns^	0.85^ns^	0.79^ns^
SR_H_	12.17^***^	9.15^**^	5.27^*^	1.31^ns^	2.52′	1.21^ns^
SR_H_/SR	8.10^***^	0.01^ns^	9.13^**^	0.13^ns^	0.84^ns^	0.79^ns^

F-values and significance codes are shown. ***P < 0.001, **P < 0.01, *P < 0.05, **′***P* < 0.1; ns indicates no significance.
