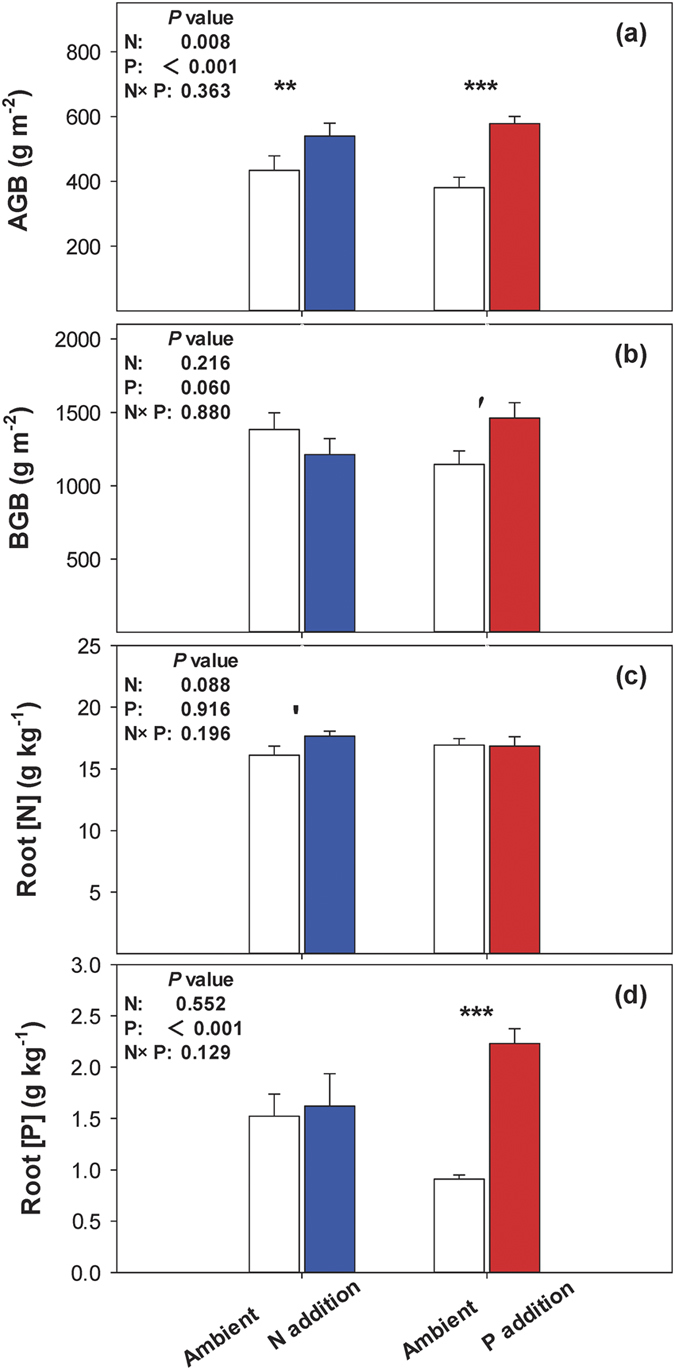# Corrigendum: Contrasting effects of nitrogen and phosphorus addition on soil respiration in an alpine grassland on the Qinghai-Tibetan Plateau

**DOI:** 10.1038/srep39895

**Published:** 2017-01-12

**Authors:** Fei Ren, Xiaoxia Yang, Huakun Zhou, Wenyan Zhu, Zhenhua Zhang, Litong Chen, Guangmin Cao, Jin-Sheng He

Scientific Reports 6: Article number: 3478610.1038/srep34786; published online: 10 10 2016; updated: 01 12 2017

In this Article, Figure 2 is duplicated as Figure 4. The correct Figure 4 appears below as Figure 1.

## Figures and Tables

**Figure 1:**