# Supplementary material for: Contrasting effects of nitrogen and phosphorus addition on soil respiration in an alpine grassland on the Qinghai-Tibetan Plateau
Source: Sci Rep. 2016 Oct 10;6:34786. doi: 10.1038/srep34786 (PMC5056390; doi:10.1038/srep34786)

Contrasting effects of nitrogen and phosphorus addition on soil respiration in an alpine grassland on the Qinghai-Tibetan Plateau

Fei Ren[1, 2, #], Xiaoxia Yang[1, 3, 4 #], Huakun Zhou[1] , Wenyan Zhu[1], Zhenhua Zhang[1], Litong Chen[1], Guangmin Cao[1] and Jin-Sheng He[1]

[1]*Key Laboratory of Adaptation and Evolution of Plateau Biota, Northwest Institute of Plateau Biology, Chinese Academy of Sciences, 23 Xinning Rd., Xining 810008, China.*

[2]*University of Chinese Academy of Sciences, 19A Yuquan Rd., Beijing 100049, China*

[3]*Qinghai Academy of Animal and Veterinary Sciences, 1 Weier Rd., Xining 810016, China*

[4]*State Key Laboratory of Plateau Ecology and Agriculture, Qinghai University, 251 Ningda Rd., Xining, 810016, China.*

[#] ***These authors contributed equally to this work.***

Supplementary Table 1 Results of mixed-effects model for repeated-measures ANOVA on effects of nitrogen (N) and phosphorus (P) additions over time (measuring date) on soil temperature and soil moisture at 5cm depth across 2009 to 2012. Significant differences (P < 0.05) are bolded and italicized. Shown are the F-values with significant codes.

	Soil temperature at 5cm depth		Soil moisture at 5cm depth	
Source	d.f.	F-value	d.f.	F-value
N	1,9	4.242^{ns}	1,9	0.349^{ns}
P	1,9	2.704^{ns}	1,9	1.799^{ns}
N × P	1,9	1.827^{ns}	1,9	0.007^{ns}
Time	3,602	36.269***	3,555	64.060***
N × Time	3,602	0.132^{ns}	3,555	0.245^{ns}
P × Time	3,602	0.219^{ns}	3,555	0.070^{ns}
N × P × Time	3,602	0.289^{ns}	3,555	0.024^{ns}

*** P < 0.001, ns, no significant.

Supplementary Table 2 Summary of soil physiochemical characteristics at different soil depths in the study site. Means of pH values, total organic carbon (TOC), total nitrogen (TN), total phosphorous (TP) and available phosphorous (AP) are shown with standard error in parentheses.

Depth	pH	TOC (g kg^{-1})	TN (g kg^{-1})	TP (g kg^{-1})	AP (mg kg^{-1})
0-5 cm	7.46 (0.04)	72.4 (0.16)	8.7 (0.12)	0.84 (0.006)	6.2 (0.009)
5-10 cm	7.61 (0.03)	62.4 (0.25)	6.9 (0.08)	0.69 (0.006)	5.2 (0.008)
10-20 cm	7.96 (0.02)	42.8 (0.16)	5.9 (0.07)	0.56 (0.018)	3.2 (0.008)
20-40 cm	8.36 (0.01)	33.0 (0.19)	3.7 (0.07)	0.44 (0.006)	2.2 (0.011)
40-60 cm	8.57 (0.02)	23.6 (0.11)	1.9 (0.06)	0.30 (0.011)	0.2 (0.006)

Supplementary Fig. 1 Dependence of soil respiration on (**a**) soil temperature at the depth of 5 cm; and (**b**) volumetric soil moisture at the depth of 5 cm in control plots across growing seasons from 2009 to 2012.

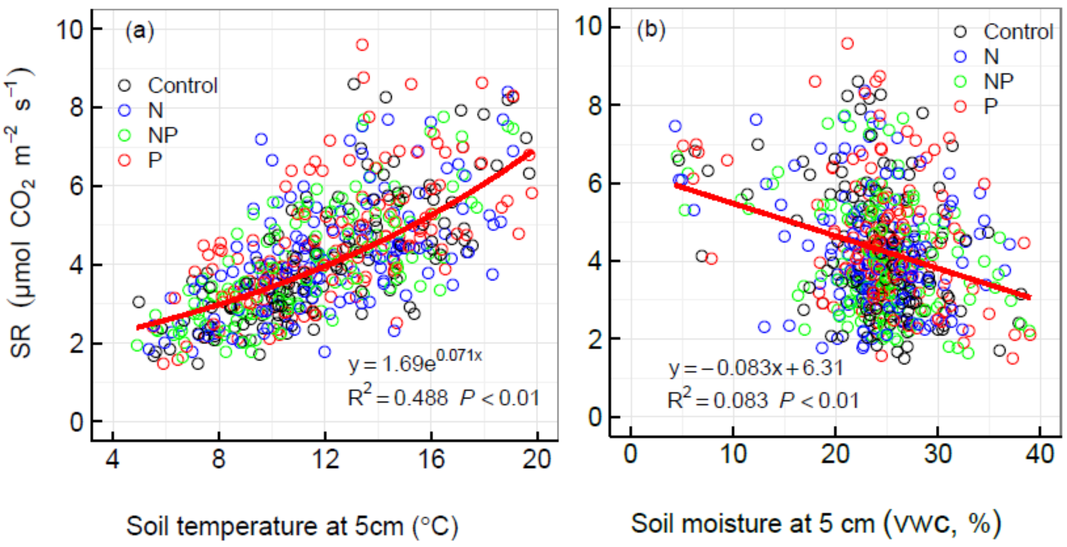

Supplement: Supplementary Information [file srep34786-s1.pdf]
